# Popper and the Omics

**DOI:** 10.3389/fpls.2016.00195

**Published:** 2016-02-19

**Authors:** Robert Winkler

**Affiliations:** Laboratory of Biochemical and Instrumental Analysis, Department of Biotechnology and Biochemistry, CINVESTAV Unidad IrapuatoIrapuato, Mexico

**Keywords:** epistemology, scientific method, inductive reasoning, data mining, model building, data integration, predictive models, knowledge creation

Omics techniques produce information, but not necessarily scientific knowledge. Genomics, Transcriptomics, Proteomics, Metabolomics, and other -omics generate unprecedented amounts of experimental data about cells or tissues under certain conditions. However, from an epistemological point of view, merely fitting data into a model to explain observations is not sufficient; science should strive to describe simple and logical theoretical systems that are testable and that enable predictions (Popper, 1959). This paper tries to alleviate this dilemma by suggesting data mining strategies to support the conversion of Omics data into resilient models.

Inductive science, which draws conclusions from empirical observations, is descriptive, and multiple models can explain the same set of data. The prediction of future events from past observations might be plausible and could appear to be in agreement with our experiences, but the derivation of natural laws or theories cannot be justified by fitting observations into a model. This “problem of induction” was introduced by Hume in the eighteenth century (Hume, 1748) and is generally accepted in epistemology.

Popper illustrates this fundamental problem in the theory of knowledge creation with his famous example of white and black swans: “Now it is far from obvious, from a logical point of view, that we are justified in inferring universal statements from singular ones, no matter how numerous; for any conclusion drawn in this way may always turn out to be false: no matter how many instances of white swans we may have observed, this does not justify the conclusion that all swans are white” (Popper, 1959).

In contrast, deductive science begins with a hypothesis or theory and proceeds to derive possible conclusions and statements that are testable, either logically or experimentally. Since it might be impossible to verify truth, even assuming infinite data, Karl Popper suggested the concept of falsifiability (Popper, 1959): Instead of collecting evidence in support of a certain hypothesis, the borders of the validity of a hypothesis are systematically explored by testing its possible theoretical consequences. This strategy is known as “The Scientific Method.”

The starting point for formulating a scientific hypothesis is usually an idea that gives a fresh and surprising view on reality. Whereas the evaluation of a new theory is a strictly systematic process, the generation of a hypothesis depends on the creativity and intuition of the researcher.

However, Omics projects usually start with a biological question or a medical problem (see Figure 1). For example, one may wish to investigate the physiological changes of an organism under suboptimal conditions or during a pathological process. Since, in comparative studies, the individuals within a sample group are representatives of a certain treatment or phenotype, stating a null hypothesis (i.e., assuming there is no difference between the groups) is not appropriate. In the context of epistemology, the conclusions drawn from such an exploratory Omics experiment would only have descriptive meaning. However, the obtained data could stimulate the formulation of hypotheses or theories (Weckwerth, 2003), which could then be tested in subsequent experiments for verification or falsification.

**Figure 1:**
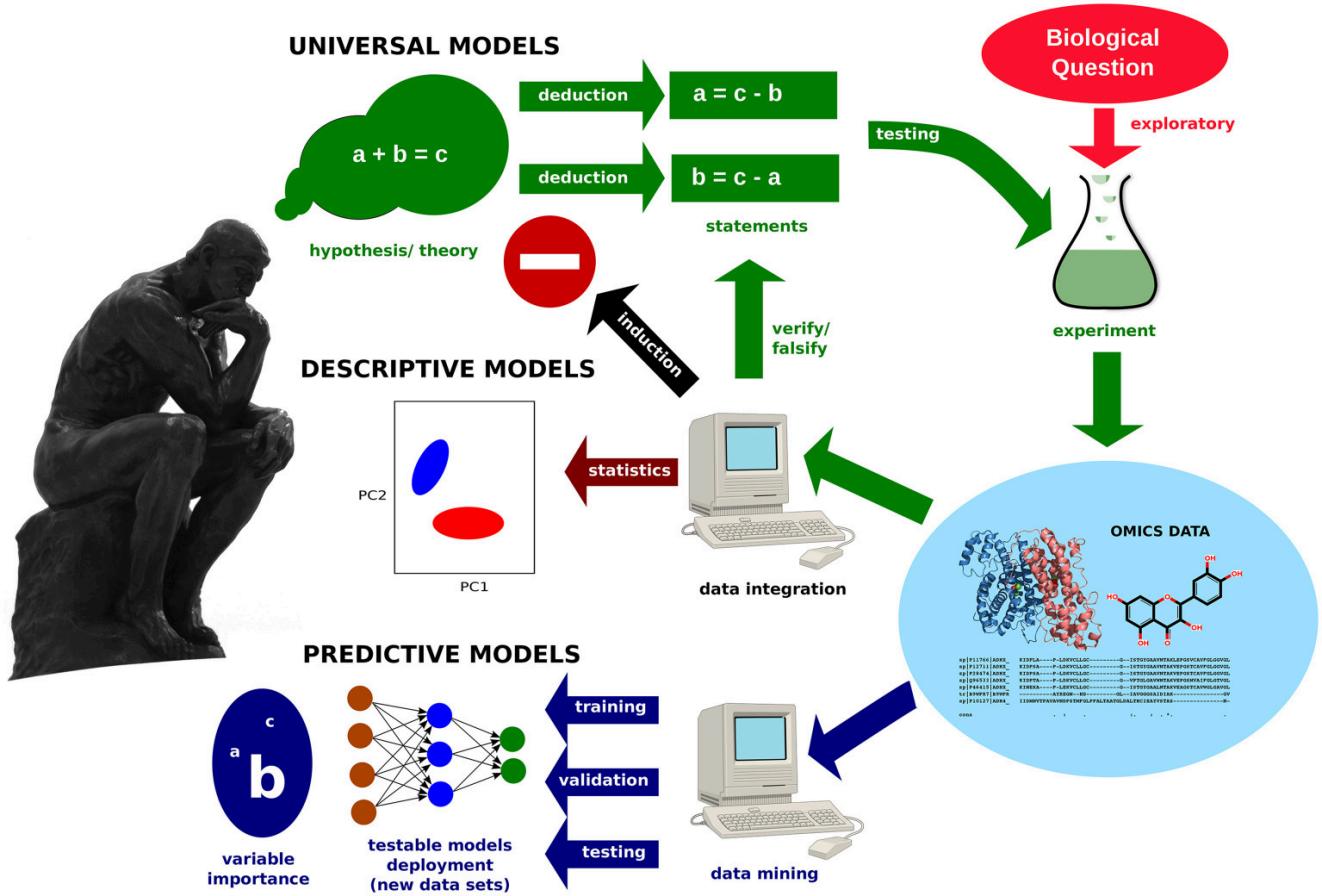
**Model development in Omics**. Exploratory studies generate data, which can be integrated into descriptive models by statistics methods. Data mining methods allow for the building of predictive models. Furthermore, important variables, as well as hidden relationships, are revealed. The creation of universally valid models requires the systematic testing of a hypothesis.

Someone might protest that life's physiological processes are too complex to be wedged into simple, testable statements. Take the theory of evolution, for instance. Following the principles of the scientific method seems impossible in this case, since the study of dynamic ecosystems would be necessary. However, there is strong evidence that Darwin himself used a hypothesis-driven approach, although giving the public impression that he followed an empirical, inductive methodology in his famous work “On the Origin of Species by Natural Selection” (Darwin, 1859; Ayala, 2009). He used this trick to ensure that his theory would be more readily accepted by the scientific community, which was evidence-focussed at that time. Darwin's strategy is well documented in a letter written in 1865 to the Scottish botanist John Scott: “Let theory guide your observations, but till your reputation is well established be sparing in publishing theory. It makes persons doubt your observations” (Darwin Correspondence Project, letter: 4206^1^). Popper initially declared the theory of natural selection as “a most successful metaphysical research program.” Later, he accepted the experimental testability of natural selection, thus confirming Darwin's theory to be congruent with the scientific method (Popper, 1978).

Classic statistics methods, such as Student's *t*-test (Student, 1908), principal components analysis (PCA) (Hotelling, 1933) or hierarchical clustering (HCA) (Ward, 1963) are helpful to extract information from data sets and to prove the significance of differences between sample sets. But, since all values of a measurement series are taken into account for a statistical analysis, speculating on the outcome of future experiments is questionable.

The data mining approach incorporates artificial intelligence and machine learning into statistics, and supports the recognition of patterns within massive data sets. Contrary to traditional statistics, only a partition of the available data is used to train data mining models. The performance of the models during optimization is monitored using an alternate partition of the data, the validation data set. Finally, the error rate of the model is estimated with the remaining data partition (Williams, 2011). Importantly, the testing data were not used when building the model, and thus represent a realistic assessment of the model's correctness when applied to new data sets.

Data mining models are only valid in a certain numerical space, but they do provide a semi-automated solution to develop models with predictive power. Further, they deliver an unbiased view on variable importance and thus support the scientist in the creation of hypotheses.

The first reports on employing data mining tools for Omics data sets appeared about 10 years ago (Truong et al., 2004; Horvatovich et al., 2006; Lin et al., 2006). Nowadays, the implementation of data mining tools in open source software with a graphical user interface, such as Rattle (Williams, 2009) and MetaboAnalyst (Xia et al., 2009), make it fairly easy to employ them in Omics workflows. Further, the predictive models have immediate utility, for example in medical diagnostics or in the classification of organisms.

Recently, we investigated the analysis of proteomics and metabolomics data using current data mining software. Random forest tree models (Williams, 1987) demonstrated excellent performance for the classification of Arabidopsis accessions and tissue types based on un-targeted metabolomics data (Sotelo-Silveira et al., 2015), and groups of chickens with different treatments (Ernest et al., 2012) could be discriminated reliably from targeted metabolomics data. It is noteworthy to point out that this separation was not possible when using clustering methods (Winkler, 2015). Additionally, the variable importance values, which are calculated during model building, point toward metabolites and pathways that are relevant for classification.

Furthermore, we demonstrated the application of association analyses in proteomics. Association analyses unveil relationships between variables and are heavily used in social media and shopping platforms (Williams, 2011). The detection of co-occurring peptides and proteins supports the discovery of protein interactions and alternative biomarkers (Winkler, 2015).

Independently of the initial experimental design, data mining methods are extremely useful for the disclosure of hidden information and surprising correlations in Omics datasets. The re-evaluation of previously collected data or public databases by data mining methods supports new discoveries and robust predictive models. Universal models, however, must be based on hypotheses that are built from theoretical considerations and that can withstand thorough, continuous testing.

## Author contributions

The author confirms being the sole contributor of this work and approved it for publication.

## Funding

I would like to thank CONACYT basic science grant I0017/CB-2010-01/151596, FINNOVA I010/260/2014 and the CINVESTAV.

### Conflict of interest statement

The author declares that the research was conducted in the absence of any commercial or financial relationships that could be construed as a potential conflict of interest.

## Footnotes

^1^Darwin Correspondence Project, letter: 4206 Available online at: (http://www.darwinproject.ac.uk/letter/entry-4206.) (Accessed July 29, 2015).
